# Evaluate the Quality of Life in Patients With Percutaneous Coronary Intervention Versus Coronary Artery Bypass Graft

**DOI:** 10.7759/cureus.52645

**Published:** 2024-01-20

**Authors:** Ashiqur Rahman, Rina Haider, Habiba Shirin, Arif Sobhan, KATM Ehsanul Huq

**Affiliations:** 1 25 Field Ambulance, Bogra Cantonment, Bogura, BGD; 2 Obstetrics and Gynecology, Square Hospitals Ltd, Dhaka, BGD; 3 Graduate School of Biomedical and Health Sciences, Hiroshima University, Hiroshima, JPN; 4 Epidemiology and Public Health, Independent Researcher, Toronto, CAN

**Keywords:** bangladesh, coronary artery disease (cad), coronary artery bypass grafting (cabg), percutaneous coronary intervention (pci), quality of life (qol)

## Abstract

Introduction

Globally, coronary heart disease is the most imperative cause of premature death. However, timely management with percutaneous coronary intervention (PCI) or coronary artery bypass graft (CABG) can improve the quality of life (QoL) and reduce mortality. The objective of this study was to evaluate the QoL between the patients who received PCI and CABG* *for the treatment of coronary heart disease.

Materials and methods

This was a retrospective observational study. Patients who underwent PCI or CABG at least three months before or more at enrollment were purposefully selected.

Results

A total of 156 patients were enrolled, 78 in each group. Health-related QoL was assessed by using the SF-36 scale for PCI or CABG procedures. The mean ± SD scores of QoL for PCI and CABG were 78.95 ±10.14 and 78.17 ± 10.92, respectively. Of the patients, 72.43% felt better after treatment, 17.95% felt the same as before treatment, and 9.62% felt worse than before treatment in both groups. Among CABG patients, 38.46% felt significantly better after treatment compared to PCI (33.97%) (p=0.048). Moreover, more CABG patients (6.41%) felt significantly worse than PCI patients (3.21%) after treatment (p=0.048). Male patients were significantly more in the CABG group (89.74%) compared to the PCI group (75.64%). In contrast, female patients had more PCI (24.36%) compared to CABG patients (10.26%) (p=0.020).

Conclusion

Subjective perceptions of physical and mental well-being improved significantly from before treatment to at least three months after treatment, and an enhanced health-related QoL was noticed for medical intervention (PCI) and surgical approach (CABG).

## Introduction

Globally, cardiovascular disease (CVD) is the leading cause of death [1,2]. It remains one of the most deadly diseases that causes disability and death in many developed countries [3]. It is alarming that due to disease transition and economic growth, CVDs are also increasing in developing countries [4]. Due to the high cost of treatment facilities, low‐ and middle‐income countries (LMICs) face a growing burden of mortality attributable to CVD. Additionally, low availability of treatment facilities including specialized hospitals, specialists, instruments, and drugs are the key barriers to the use of and adherence to essential treatments of CVD. As a preventive measure for CVD, the use of antihypertensive medications is rather expensive worldwide, particularly for LMICs [5]. Moreover, for patients suffering from CVD at older ages, the presence of comorbidities makes the treatment fragmented and uncoordinated and makes the treatment decisions more complex [6].

Coronary artery disease (CAD) is caused by the accumulation of atheromatous plaques within the walls of the coronary arteries. To treat CAD, percutaneous coronary intervention (PCI) and coronary artery bypass grafting (CABG) are considered revascularization procedures. However, there is no clear-cut indication of which procedure, PCI or CABG, would lead to better treatment outcomes [7,8]. Moreover, it is difficult to predict the treatment effects of PCI and CABG due to the viability of blood supply through cardiac vessels and/or ischemic detection to guide revascularization, which might be an influencing factor in choosing the better treatment option [9].

PCI is the medical intervention and CABG is the surgical approach for the treatment of CAD. Heart-team physicians recommend suitable procedures (PCI or CABG) for the patients by considering the complexity of the coronary disease, comorbidities, surgical risk, patients’ preferences, and hospital culture [10].

The perception of quality of life (QoL) depends on the individual’s culture and values where they live and related to their goals, expectations, standards, and concerns [11]. QoL is considered a significant concept and outcome in healthcare. To evaluate health-related QoL (HRQoL), we can identify the physical or mental health status of the individuals and can improve their health through interventions [12]. QoL is a factorial structure that consists of five dimensions: (i) physical and material well-being, (ii) relationships with other people, (iii) social, community, and civic activities, (iv) personal development and fulfillment, and (v) recreation. To assess QoL, we can evaluate the level of satisfaction of individuals with these aspects of life [13]. There are several instruments existing to assess the QoL, especially for patients with CAD. To assess the QoL of cardiac patients, a 36-item Short Form Health Survey (SF-36) questionnaire is one of the most widely common health status instruments used extensively [14].

This study aimed to increase understanding of the characteristics of illness and implications of HRQoL to measure and compare post-PCI or CABG life, to determine factors that influence the QoL.

## Materials and methods

This was a retrospective observational study conducted between January 01, 2019, and December 31, 2019. The study was conducted at the National Institute of Cardiovascular Diseases (NICVD), Ibrahim Cardiac Hospital and Research Institute (ICHRI), Bangabandhu Sheikh Mujib Medical University (BSMMU), and the National Heart Foundation Hospital and Research Institute in Dhaka, Bangladesh. These referral hospitals are the main cardiac hospitals in Bangladesh and were selected with the expectation of getting large samples and different treatment approaches. The study was reviewed and approved by the independent Ethical Committee of the National Institute of Preventive and Social Medicine, Bangladesh (approval number: NIPSOM/EC/2019/1406). Written informed consent was taken from all the participants before enrollment.

Inclusion and exclusion criteria

All patients who had a history of PCI or CABG procedure at least three months or more prior to the study attended the cardiac medicine and surgery outpatient department for their review and follow-up, and were willing to participate in this study were included. Patients who developed postoperative complications or co-morbidities such as stroke, chronic obstructive pulmonary disease, bronchial asthma, or chronic renal failure, and were unable to respond to the study procedures were excluded.

Data collection and procedures

**Figure 1:**
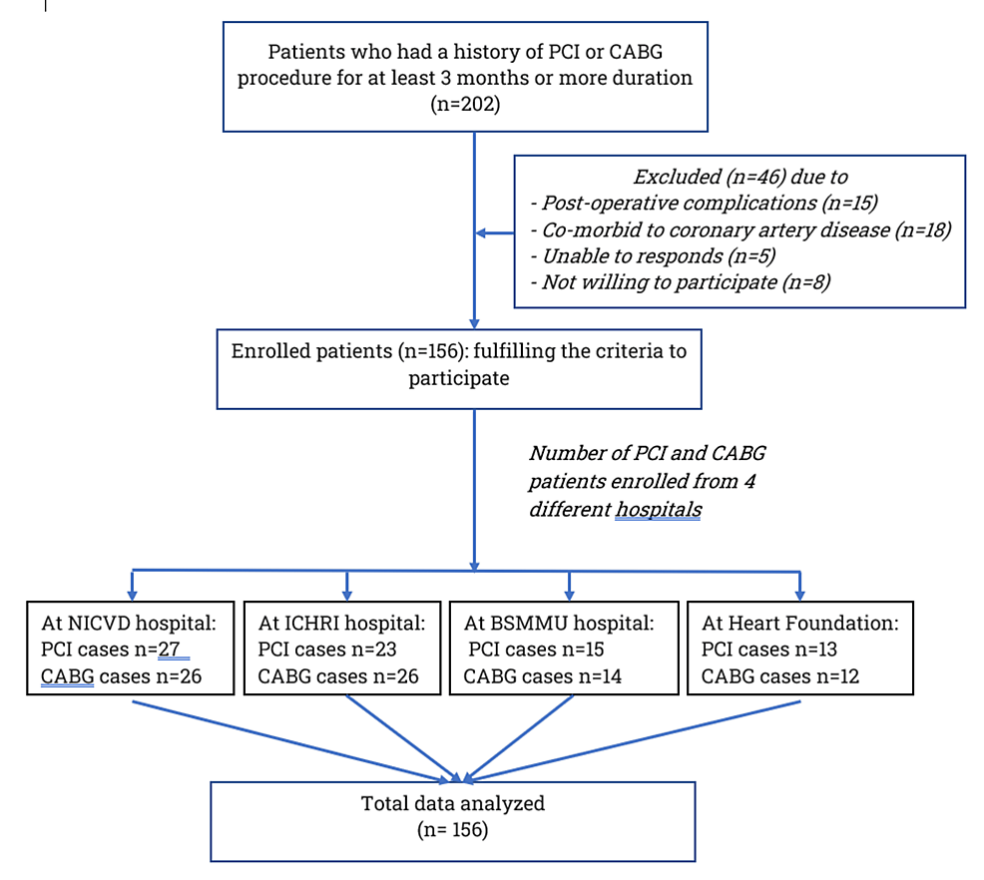
Flow chart of study participants. NICVD: National Institute of Cardiovascular Disease and Hospital; ICHRI: Ibrahim Cardiac Hospital and Research Institute; BSMMU: Bangabandhu Sheikh Mujib Medical University; PCI: percutaneous coronary intervention; CABG: coronary artery bypass graft

The data was collected simultaneously from four different hospitals mentioned above. Researchers visited hospital outpatient departments every weekday and checked the eligible criteria. After confirming the inclusion and exclusion criteria, they obtained consent from the patients and filled up the questionnaires in Bengali. A face-to-face interview was conducted anonymously and as confidentially as possible. The variables selected were socio-demographic data, characteristics of illness, clinical investigations and treatment approach, and determinants of QoL. Figure 1 shows the selection process.

Sample size calculation

Using the StatCalc of Epi Info™ Version 7 for Windows (Centers for Disease Control and Prevention, Atlanta, Georgia, United States), assuming the expected population size was 100 for each group (PCI or CABG), the frequency of enrollment was 30%, and the margin of error was 4.8% with a 95% confidence interval, the estimated sample size was 78 for PCI and 78 for CABG, and the total sample size was 156.

Measurement of health status

Medical outcomes study SF-36, which was derived from the work of the Rand Corporation (Santa Monica, California, United States), was used to measure self-reported physical and mental health status (QoL) [14]. This is a generic, multi-dimensional tool that consists of 36 questions (items) on eight health concepts. Physical components comprise physical functioning, role physical, bodily pain, and general health. Mental components consist of vitality, social functioning, role emotional and mental health. Responses to each item were scored and summed according to a standardized scoring protocol and expressed as a score on a 0-100 scale for each of the eight health concepts. A higher score indicates a better QoL [15]. Therefore, by using this tool this study assessed the physical, social, functional, and emotional well-being of every individual who had undergone PCI or CABG procedure. 

We applied the SF-36 Bengali version in our study. This Bengali SF-36 was used among the patients of Bangladesh to see the acceptability, reliability, and validity and was found valid and reliable (Cronbach’s α >0.78 and reliability r>0.82) for all scales [16]. 

Statistical analysis

Descriptive statistics were expressed as frequencies and percentages for categorical variables and mean and standard deviation for continuous variables. To compare the categorical variables between two groups, the chi-square test, and for continuous variables, the two-sided t-test or Mann-Whitney U test were performed as appropriate. The significance level was set at a P-value of <0.05. We analyzed data using IBM SPSS Statistics for Windows, Version 21.0 (Released 2012; IBM Corp., Armonk, New York, United States).

## Results

A total of 156 patients were enrolled and data were analyzed for PCI (n=78; 50%) or CABG (n=78; 50%) groups from four different hospitals.

Socio-demographic characteristics

The mean±SD age of the participants was 59.49 ± 11.29 years with a range of 40-92 years. The average age for the PCI group was 58.10±10.34 years and CABG was 60.88±12.07 years. Male participants were more in the CABG (n=70; 89.74%) than PCI (n=59; 75.64%) group. The majority of the patients were married (n=143; 91.66%) and Muslim (n=149; 95.51%) in religion. There was no significant difference (p=0.219) for the up to higher secondary (25.64% vs. 24.36%), Bachelor's degree (12.82% vs. 15.38%), and above Bachelor's degree (11.54% vs. 10.26%) level of education between PCI and CABG groups. The occupation of the majority of participants in both groups was business (PCI: n=26, 16.67%; CABG: n=32, 20.51%), followed by service (PCI: n=19, 12.18%; CABG: n=22, 14.1%), other formal and informal occupation (PCI: n=17, 10.90%; CABG: n=18, 11.54%) and housewife (PCI: n=16, 10.26%; CABG: n= 6, 3.8%). The average monthly expenditures for PCI and CABG participants were $ 318.23±159 and $ 334.96±202, respectively (Table 1). 

**Table 1:** Comparison of socio-demographic and health-related characteristics in PCI and CABG-treated patients p<0.05 considered as significant QoL: quality of life; PCI: percutaneous coronary intervention; CABG: coronary artery bypass graft

Characteristics	Total QoL	PCI (n=78)	CABG (n=78)	p-value
Age (years), mean±SD	59.49±11.29	58.10±10.34	60.88±12.07	0.297
Sex, n	156	78	78	0.020
Male, n (%)	129 (82.69)	59 (75.64)	70 (89.74)	
Female, n (%)	27 (17.31)	19 (24.36)	8 (10.26)	
Religion				0.013
Muslim, n (%)	149 (95.51)	72 (46.15)	77 (49.36)	
Non-muslim, n (%)	07 (4.49)	06 (3.8)	01 (0.64)	
Marital status				0.517
Married, n (%)	143 (91.66)	71 (45.51)	72 (46.15)	
Unmarried, n (%)	01 (0.64)	00 (00)	01 (0.64)	
Widowed, n (%)	12 (7.7)	07 (4.49)	05 (3.21)	
Educational Level				0.219
Up to higher secondary (≤12 years), n (%)	78 (50.0)	40 (25.64)	38 (24.36)	
Bachelor’s degree (13-15 years), n (%)	44 (28.2)	20 (12.82)	24 (15.38)	
Above bachelor’s degree (≥15 years), n (%)	34 (21.8)	18 (11.54)	16 (10.26)	
Occupation				0.410
Service, n (%)	41 (26.28)	19 (12.18)	22 (14.10)	
Business, n (%)	58 (37.18)	26 (16.67)	32 (20.51)	
Housewife, n (%)	22 (14.10)	16 (10.26)	06 (3.8)	
Others (formal and informal), n (%)	35 (22.44)	17 (10.90)	18 (11.54)	
Monthly expenditure (USD), mean ±SD		318.23 (159)	334.96 (202)	0.568
Angiogram Report, n (%)	132 (84.61)	65 (41.67)	67 (42.95)	0.127
Single vessel block, n (%)	42 (26.92)	26 (16.67)	16 (10.26)	
Double vessel block, n (%)	60 (38.46)	27 (17.31)	33 (21.15)	
Triple vessel block, n (%)	30 (19.23)	12 (7.69)	18 (11.54)	
Duration of suffering from symptoms to treatment (days), mean±SD		87.27±12	122.92±16	0.067
Post-treatment period (months), mean±SD		25.23±20.94	18.27±16.81	0.024
Health state after the treatment				0.048
Feel better after the treatment, n (%)	113 (72.43)	53 (33.97)	60 (38.46)	
Same as before the treatment, n (%)	28 (17.95)	20 (12.82)	8 (10.26)	
Worse than before the treatment, n (%)	15 (9.62)	05 (3.21)	10 (6.41)	

Characteristics of illness

The average duration of suffering from diagnosis to treatment was 87.27±12 days for PCI and 122.92±16 days for CABG and the difference was not significant (p=0.067). The post-treatment duration from PCI/CABG procedure to data collection was 25.23±20.94 months for PCI and 18.27±16.81 months for CABG groups and it was found significant (p=0.024). According to angiogram reports, the proportion of single vessel blocks was higher (16.67% vs. 10.26%) among PCI-treated cases. In contrast, double (17.31% vs. 21.15%) and triple (7.69% vs. 11.54%) vessel blocks were more common among CABG patients compared to the PCI group, respectively, and the difference was not significant (p=0.127). The majority (n=113; 72.43%) of the participants felt better after their treatment and it was comparatively higher among CABG (n=60; 38.46%) than PCI-treated patients (n=53; 33.97%) (p=0.048). However, the proportion of participants feeling worse was higher among the CABG-treated group (n=10; 6.41%) compared to their counterparts (n=5; 3.21%) (Table 1).

HRQoL

**Figure 2:**
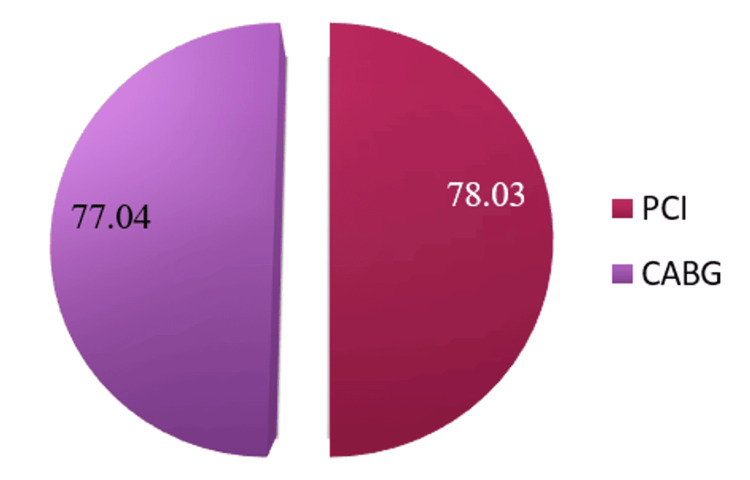
Distribution of physical component scores of quality of life. p=0.618, (p<0.05 considered as significant) PCI: percutaneous coronary intervention; CABG: coronary artery bypass graft

**Figure 3:**
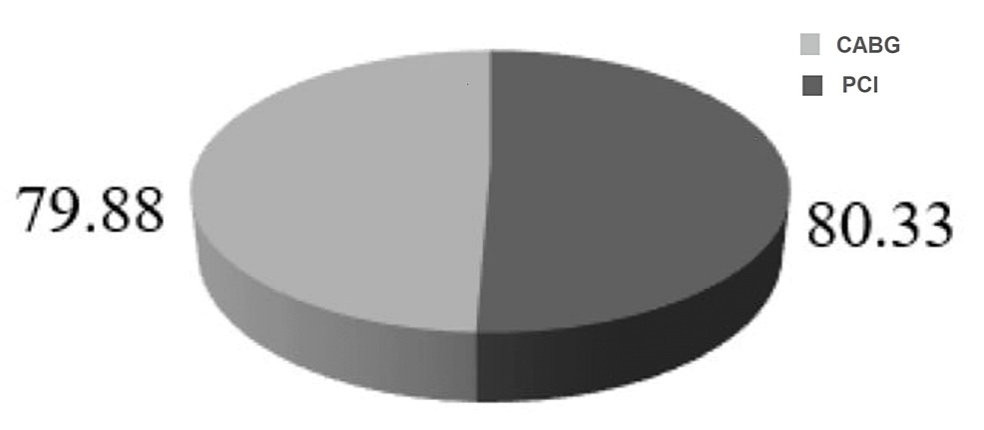
Distribution of mental component scores of quality of life. p=0.783, (p<0.05 considered as significant) PCI: percutaneous coronary intervention; CABG: coronary artery bypass graft

**Figure 4:**
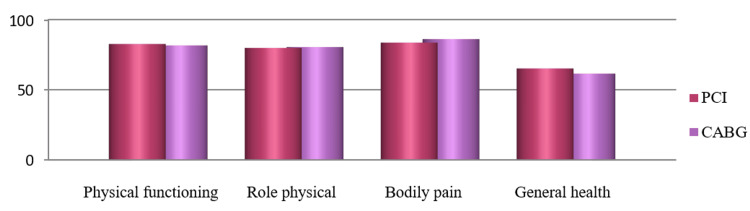
Distribution of mean value of various domains of the physical components of quality of life.

**Figure 5:**
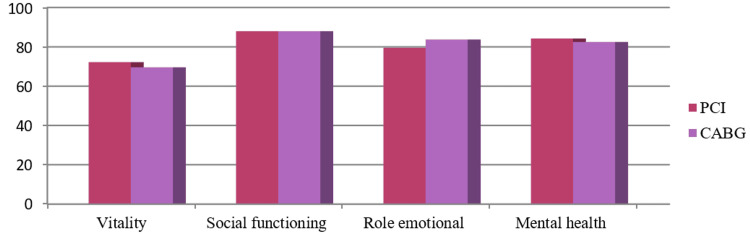
Distribution of mean value of various domains of the mental component of quality of life.

The mean±SD of the total score of QoL generated from all eight domains were 78.95±0.14 and 78.17±10.92 for PCI and CABG, respectively. The average score of physical components was 78.03±12.47 for PCI and 77.04±12.38 for CABG, and the average score of mental components was 80.33±9.66 for PCI and 79.88±11.03 for CABG. As the numerical values for QoL between the PCI and CABG groups are commensurate with each other, the difference between PCI and CABG in total QoL and physical, mental, and individual components was found not significant (Table 2, Figures 2-5). 

**Table 2:** Comparison between treatment (PCI/CABG) groups according to scores of individual domains and QoL p<0.05 considered as significant PCI: percutaneous coronary intervention; CABG: coronary artery bypass graft; QoL: quality of life

Components	Treatment group	Mean ± SD	t test	p-value
Physical component	PCI	78.03± 12.47	0.499	0.618
	CABG	77.04± 12.38		
Physical functioning	PCI	82.63±13.54	0.484	0.629
	CABG	81.61± 12.49		
Role physical	PCI	79.81±21.34	-0.160	0.873
	CABG	80.44± 28.10		
Bodily pain	PCI	83.62 ± 22.07	-0.870	0.421
	CABG	86.09±15.57		
General health	PCI	65.19± 17.86	1.236	0.799
	CABG	61.53 ±19.04		
Mental component	PCI	80.33± 9.66	0.485	0.783
	CABG	79.88± 11.03		
Vitality	PCI	72.24±15.26	1.015	0.312
	CABG	69.61±17.03		
Social functioning	PCI	87.98 ±13.87	0.00	1.000
	CABG	87.98 ±14.86		
Role emotional	PCI	79.49 ±22.94	-1.098	0.274
	CABG	83.76 ±25.61		
Mental health	PCI	84.26 ±10.75	1.029	0.305
	CABG	82.51±10.40		
Total score of quality of life	PCI	78.95 ±10.14	-3.92	0.645
	CABG	78.17 ±10.92		

## Discussion

This study was conducted to compare the outcomes in terms of QoL amongst real-world patients undergoing PCI or CABG treatment procedures. Comparatively higher scores of QoL were found in the case of PCI compared to CABG patients; however, no significant difference was found in the total scores of QoL for PCI and CABG patients.

A study by Abdallah et al. observed that patients who underwent CABG had better QoL between six months and two years compared to those who underwent PCI; however, these differences were not found significant [17]. Another study revealed that patients who underwent PCI experienced significantly higher HRQoL in six months after revascularization compared to CABG [18]. However, continuing over 24 months of follow-up, no significant differences were found between the two groups. Regarding the improvement of QoL in both groups, most patients felt better after treatment, and only a few patients felt the same and worse than before treatment. Similar results were found in another study conducted by Singh et al. in India [19]. In contrast, more CABG patients felt significantly worse than PCI patients after treatment. As CABG treatment is mostly done for double and triple vessel blocks compared to single vessels [20], they might feel better after more suffering. However, a few of the patients felt worse and might be suffering from other comorbidities. CABG patients have a much higher incidence of atrial fibrillation, which usually adds to the worse QoL compared to PCI [21-23]. Moreover, CABG predicted lower improvements in overall QoL compared to PCI [24]. In contrast, another comparative study observed QoL of PCI-treated patients (48.3%) was higher than CABG (47.6%) after six months of treatment [25]. Moreover, there is a very low incidence of stent thrombosis and in-stent restenosis (ISR) and the need for repeat interventions in the PCI group owing to the coming of biodegradable polymer drug-eluting stents (DES) [26,27].

In the current study, it was found that male patients had significantly more CABG compared to PCI. In contrast, female patients had significantly more PCI compared to CABG. A study showed that 67% of PCI and 72% of CABG patients were men, which is similar to our study [8]. Though all patients irrespective of their sex who attended the study hospitals were enrolled, male patients were about five times higher; moreover, male patients in the CABG group were three times higher compared to female patients. Another study also had similar findings with much higher numbers of male participants (76.5%) [28]. One study observed that QoL became lower, and improvement of physical health was much slower for women after CABG surgery compared to male patients [29]. This finding might influence cardiac surgeons to perform less CABG on female patients. However, it needs more investigations to explore the factors for the participation of more male patients and performing more CABG in different studies.

The mean age of the participants in the current study was 59.49 years with a range of 40-92 years; for the PCI group, it was 58.10 years, and for CABG it was 60.88 years. This indicates that CAD is associated with the older age group. It corresponds with the finding of another study where the mean age ranged from 56.9 to 74 in the PCI group and 53 to 75.2 years in the CABG group [8]. The findings also support that elderly patients had complications with more cardiovascular disease risk factors [30]. The majority of the patients in the current study were married and were Muslim in religion. A study on the quality of post-primary PCI patients’ lives showed that most participants were males and married [31].

In the present study, there was no significant difference observed regarding participants’ level of education in the PCI and CABG groups. However, a study in Iran found statistically significant differences in the level of education between the two groups [18]. Participants who had more than six grades of education experienced better HRQoL compared to those who had six or fewer grades of education. The occupation of most participants in both groups was business, followed by service, formal and informal occupation, and housewife. CVD can be influenced by occupation and an individual’s lifestyle. One study revealed a significant association between occupational stress levels and coronary heart disease; the high-income group had a higher proportion of CAD [32]. In participants of the current study, the average monthly expenditure for PCI and CABG were $318.23 and $334.96, respectively. A study conducted in Brazil, a middle-income country, found that direct costs were not different between CABG and PCI patients with a median of $630.72 and $672.29, respectively [33].

The average duration of suffering from diagnosis to treatment was 87.27 days for PCI and 122.92 days for CABG and the difference was not significant. The patient’s treatment delay might have different underlying factors. They needed to arrange treatment costs as most of the costs were borne by them out of pocket. Sometimes, they take a second opinion for treatment procedures. The patient’s mental and emotional factors also played a key role in delayed treatment. The mean (SD) waiting time was 4.1 (9.7) days from diagnostic coronary angiogram to PCI and 14.5 (25.3) days to CABG, which was not significantly associated [34]. The post-treatment duration from PCI/CABG procedure to data collection was 25.23 months for PCI and 18.27 months for CABG groups and it was found significant. According to angiogram reports, the proportion of single vessel blocks was higher among PCI-treated cases. In contrast, double and triple vessel blocks were more common among CABG patients compared to the PCI group and the difference was not significant. A study in Italy found that patients treated with PCI were more likely to have peripheral artery disease and conversely, patients treated with CABG were more likely to present with multivessel disease [20]. Another study compared 100 patients ≥55 years of age and found a very low prevalence of triple-vessel CAD in the PCI group (14.3%) as compared to 43% in the CABG group [35]. Similarly, significant left main CAD has been found in 3-5% of all patients who undergo coronary angiography and in 10-30% of patients who undergo bypass surgery [36].

Study limitations

There were certain limitations in our study. QoL is a subjective measurement and assumes patients answer how they were feeling about their life after PCI or CABG operation. There might be recall bias as few data were collected by asking history of the participants’ illness. Gender has been recognized as a significant factor in HRQoL assessments in past research. Studies using instruments like the SF-36 have often analyzed data based on gender to understand potential differences in self-reported health status between men and women. Studies also have reported variations in specific health domains between genders. For example, women may score differently than men in domains such as mental health, social functioning, and bodily pain. We used SF-36 in our study, and it was a limitation of the study method. We did not enroll any patients with reintervention; therefore, the reintervention rate and influence of QoL between groups were not determined. As a retrospective observational study, we could not compare pre- and post-operative QoL. However, we conducted this study in four different tertiary hospitals, and the results of this study might shed some light for clinicians and policymakers for the management of CVD patients. 

## Conclusions

There was a significant difference between the principal treatment procedures of PCI and CABG with participant’s sex, religion, duration from PCI/CABG procedure to data collection, and health state compared to the pre-treatment state. The total HRQoL scores for physical and mental components after getting treatment were good. As the duration of length of suffering was greater for the CABG than the PCI group, the perception of feeling better than the pre-treatment status was significantly higher in the case of CABG patients. QoL was improved and felt better after receiving treatment in both PCI and CABG-operated groups. This study's findings may help to assist further the implementation of future health interventions for heart-team physicians to make decisions for the treatment of revascularization. Further intervention studies with large sample sizes will guarantee a high probability of determining factors that influence the QoL. 
